# Ambient urban N deposition drives increased biomass and total plant N in two native prairie grass species in the U.S. Southern Great Plains

**DOI:** 10.1371/journal.pone.0251089

**Published:** 2021-05-06

**Authors:** Alexandra G. Ponette-González, Michelle L. Green, Justin McCullars, Laura Gough

**Affiliations:** 1 Department of Geography and the Environment, University of North Texas, Denton, Texas, United States of America; 2 Department of Biology, University of Texas Arlington, Arlington, Texas, United States of America; Chinese Academy of Forestry, CHINA

## Abstract

Remnants of native tallgrass prairie experience elevated atmospheric nitrogen (N) deposition in urban areas, with potential effects on species traits that are important for N cycling and species composition. We quantified bulk (primarily wet) inorganic N (NH_4_^+^-N + NO_3_^-^-N) deposition at six sites along an urban development gradient (6–64% urban) in the Dallas-Fort Worth metropolitan area from April 2014 to October 2015. In addition, we conducted a phytometer experiment with two common native prairie bunchgrass species––one well studied (*Schizachyrium scoparium*) and one little studied (*Nasella leucotricha*)––to investigate ambient N deposition effects on plant biomass and tissue quality. Bulk inorganic N deposition ranged from 6.1–9.9 kg ha^-1^ yr^-1^, peaked in spring, and did not vary consistently with proportion of urban land within 10 km of the sites. Total (wet + dry) inorganic N deposition estimated using bulk deposition measured in this study and modeled dry deposition was 12.9–18.2 kg ha^-1^ yr^-1^. Although the two plant species studied differ in photosynthetic pathway, biomass, and tissue N, they exhibited a maximum 2-3-fold and 2-4-fold increase in total biomass and total plant N, respectively, with 1.6-fold higher bulk N deposition. In addition, our findings indicate that while native prairie grasses may exhibit a positive biomass response to increased N deposition up to ~18 kg ha^-1^ yr^-1^, total inorganic N deposition is well above the estimated critical load for herbaceous plant species richness in the tallgrass prairie of the Great Plains ecoregion and thus may negatively affect these plant communities.

## Introduction

Over the past decade, there has been a surge in research on nitrogen (N) deposition in cities around the world (e.g., [1–6]). Although there are many urban areas for which data are still sparse or nonexistent [7], research highlights three distinguishing characteristics of urban N deposition. First, compared to nearby rural and remote sites, wet or bulk N deposition in urban areas is, on average, twofold higher [3]. Second, N deposition can exhibit high intra-urban (e.g., [8]) and intra-annual variability (e.g., [9]). Third, responses to urban N deposition remain poorly documented: a recent meta-analysis found that just ~10% of studies concomitantly measured N deposition and some biogeochemical metric (e.g., N mineralization) that might be affected by N [3].

Indeed, much of the current understanding of plant responses to and recovery from N addition is derived from experimental field manipulations which are most often conducted in plant communities outside the influence of urban air pollution (e.g., [10–15]). These studies show that individual plants exposed to elevated levels of N deposition often exhibit changes in biomass, resource allocation (e.g., root:shoot ratio), and tissue quality in the form of decreased carbon (C) to N ratio. In turn, lower C:N ratios have the potential to affect energy transfer to higher trophic levels by increasing the palatability of tissues to herbivores (e.g., [16]).

While informative, experimental manipulations usually involve large N pulses (e.g., >75 kg N ha^-1^ yr^-1^; [17]), whereas natural ecosystems typically experience chronic N deposition at rates well below these values [3]. However, because N accumulates within ecosystems, even lower levels of N deposition can lead to ecological changes if N deposition is elevated above background and persists over time [18–20]. The quantitative threshold above which atmospheric N deposition is expected to have negative impacts on ecosystems is termed “critical load” [21]. Observational and experimental studies show that critical load exceedances drive altered plant community composition and decreased species richness across diverse biomes (e.g., [22]), with changes often conditional on soil and climate characteristics [20]. In urban areas, N deposition effects on ecosystems are likely also contingent on local gradients in heat, rainfall, ozone, carbon dioxide, and their interactions [23].

Compared to research on N deposition impacts on urban forests [6, 24–27], urban grassland responses to N deposition are less well understood and warrant further attention. Grasslands play important roles in carbon sequestration, water quality protection, non-native species suppression, and wildlife habitat provision [28, 29]. To begin to fill this gap, we quantified bulk inorganic N––ammonium (NH_4_^+^-N) and nitrate (NO_3_^-^-N)––deposition effects on two common native prairie grass species along an urban development gradient. We hypothesized that (1) N deposition would increase with proportion of urban land (within 10 km) and (2) plant biomass would increase, while root:shoot ratio and tissue C:N would decrease, over the urban-induced deposition gradient. Our results expand current understanding of urban N deposition and prairie grass responses to rising urban N; both types of observations are sparse in the Southern Great Plains ecoregion and in numerous regions worldwide.

## Materials and methods

### Urban development and tallgrass prairie in Dallas-Fort Worth

**Fig 1:**
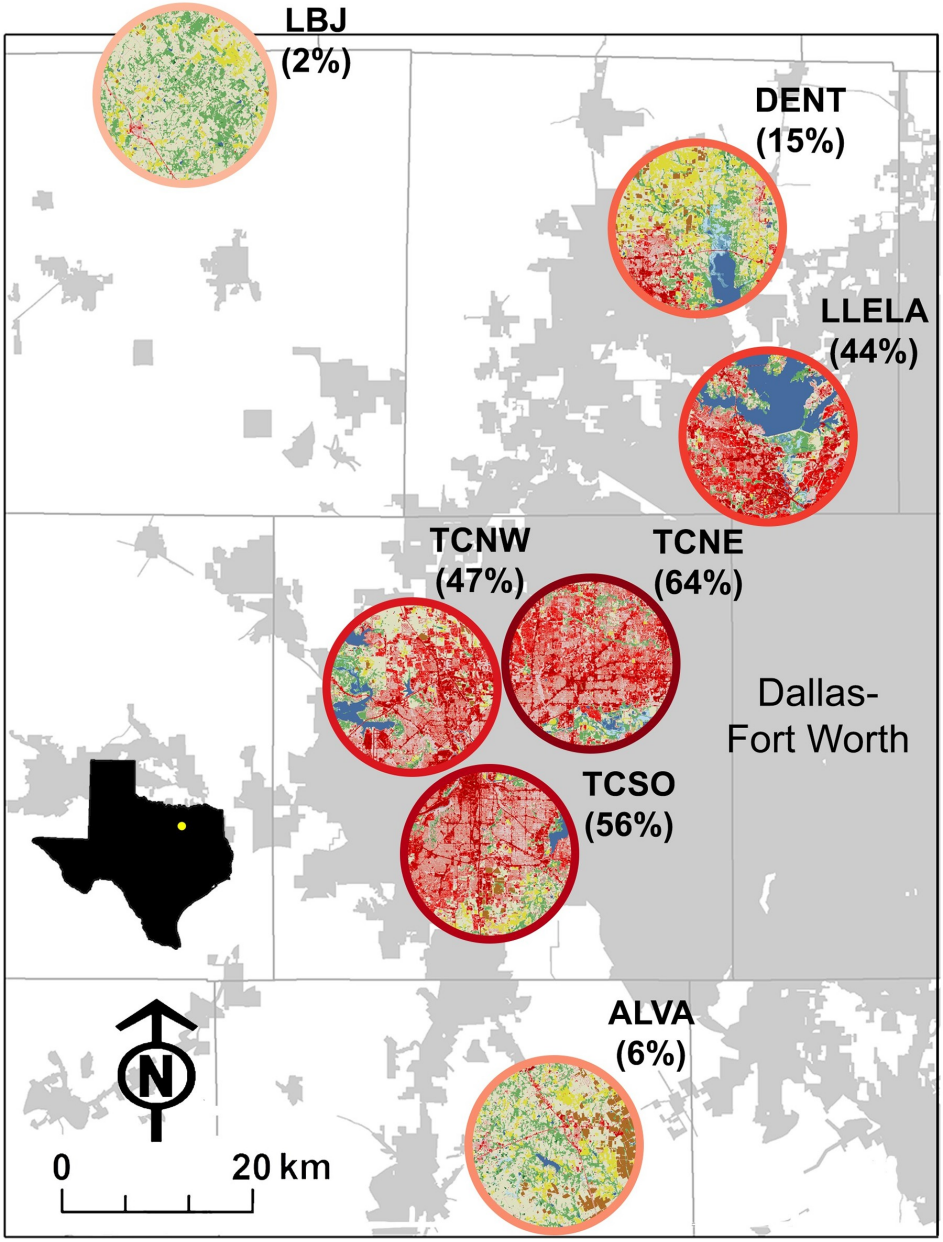
Study area map. Sites where bulk inorganic N deposition was measured in the Dallas-Fort Worth metropolitan area (municipal areas in grey) and the L.B.J. National Grasslands National Atmospheric Deposition Program (NADP) site. Percent urban development within a 10-km radius buffer [39] is indicated in parentheses and ranges from low (light pink border) to high (crimson red border).

The research was conducted in the Dallas-Fort Worth (DFW) metropolitan area, Texas (Fig 1). Settlement of DFW began in the mid-19^th^ century, with ranching as the primary economic activity [30]. The first major period of population growth in the cities of Dallas and Fort Worth occurred in the late 1880s, while significant population growth in the intermediate cities did not occur until a half century later [31]. Since the 1960s, DFW has expanded rapidly: between 2010–2018, DFW was the metropolitan area with the largest numeric population growth in the U.S. [32]. Today, it is the fourth most populous metropolitan area, with a population of >7 million [32]. Regionally, DFW is part of the greater “Texas Triangle” (Houston-Austin-San Antonio-Dallas), one of several U.S. megapolitan regions (i.e., clustered networks of cities) identified as important for research on urbanization across societal and environmental gradients [7].

Situated at the southern end of the Great Plains ecoregion, a semi-arid grassland extending from Canada through the central U.S. into Mexico, DFW straddles the Grand Prairie to the west and the Blackland Prairie to the east. Climate is humid sub-tropical with hot summers, cool winters, and intermediate rainfall (annual normal (1981–2010) ~918 mm yr^-1^; [33]). Despite rapid urbanization, considerable land areas in DFW are being used for agriculture [34], especially surrounding Tarrant and Dallas counties where the cities of Fort Worth and Dallas, respectively, are located. Together, agriculture and urbanization have resulted in the contraction of native prairie to just 1–2% of the landscape [35, 36]. Remnants of late-successional tallgrass communities––often intermixed with grazed grasslands––contain little bluestem (*Schizachyrium scoparium*), big bluestem (*Andropogon gerardii*), and Indiangrass (*Sorghastrum nutans*) [37], with little bluestem the dominant plant species [38]. Associated species include side-oats grama (*Bouteloua curtipendula*), tall dropseed (*Sporobolus compositus*), and Texas wintergrass (*Nasella leucotricha*).

### Site selection

Six sites were selected along an urban development gradient (Fig 1, Table 1) based on property owners’ willingness to participate, accessibility, and suitability to the research study. All sites were located in the Fort Worth Prairie, the northern portion of the larger vegetation unit known as the Grand Prairie [38], where pasture, grassland and herbaceous cover occur alongside and are inversely correlated with percent urban land. Three sites were located on college or county campuses with nearby buildings and groomed landscapes. These included Tarrant County College Northwest (TCNW), Tarrant County College Northeast (TCNE), and the Resource Connection of Tarrant County, a campus of county services buildings and a demonstration garden (TCSO). Two properties were located within designated natural areas with few nearby buildings and some restoration management on the landscape: Lewisville Lake Environmental Learning Area (LLELA) and Clear Creek Natural Heritage Center in Denton County (DENT). An additional agricultural site was located adjacent to a house and farm animals on private property in the community of Alvarado (ALVA). The National Atmospheric Deposition Program (NADP) monitoring site at Lyndon B. Johnson National Grasslands (L.B.J., TX56), located 78 km north-northwest of Fort Worth, was used as a non-urban reference site since NADP monitoring sites are located distant from local pollution sources. Thus, NADP measurements of wet N deposition underestimate deposition in urban-affected areas but serve as useful estimates of background deposition [6].

**Table 1:** Study site characteristics.

Site ID	Latitude, Longitude	% Urban^a^	% Crops^b^	% Pasture^c^	% Grassland^d^	Rainfall^e^	NH_4_^+^-N	NO_3_^-^-N	Inorganic N
LBJ	33.3917, -97.6397	2	<1	9	57	2048	3.2	1.9	5.0
ALVA	32.3962, -97.2395	6	11	11	48	1421	4.0	2.2	6.1
DENT	33.2606, -97.0649	15	4	20	29	1921	6.0	2.6	8.6
LLELA	33.0629, -96.9884	44	<1	1	11	1490	4.0	2.4	6.4
TCNW	32.8315, -97.3917	47	1	2	19	1161	6.2	3.7	9.9
TCSO	32.6743, -97.3082	56	2	4	12	1100	5.1	2.1	7.2
TCNE	32.8509, -97.1906	64	<1	1	7	1347	4.1	2.4	6.5

Land cover within a 10-km radius buffer [39], rainfall (mm), and bulk nitrogen deposition (kg ha^-1^ yr^-1^) for six sites along an urban development gradient in the Dallas-Fort Worth metropolitan area and the NADP L.B.J. National Grasslands network site [40]. Rainfall, bulk NH_4_^+^-N, NO_3_^-^-N, and inorganic N (NH_4_^+^-N + NO_3_^-^-N) deposition are for the period 4 April 2014 to 9 October 2015 (this study). Numbers do not always sum due to rounding.

^a^ Urban: sum of Developed High Intensity, Developed Medium Intensity, and Developed Low Intensity categories.

^b^ Cultivated crops: areas used for the production of annual and perennial woody crops. This class also includes actively tilled land.

^c^ Pasture/Hay: areas of grasses, legumes, or grass-legume mixtures planted for livestock grazing or the production of seed or hay crops.

^d^ Grassland/Herbaceous: areas dominated by graminoid or herbaceous vegetation. These areas are not subject to intensive management such as tilling but can be utilized for grazing.

^e^ Rainfall measured using tipping-bucket rain gauges at meteorological stations closest to the study sites [41].

### Nitrogen deposition measurements

Bulk inorganic N (NH_4_^+^-N + NO_3_^-^-N) deposition was measured from 4 April 2014 to 9 October 2015 at the six sites described above in open areas with no canopy cover and >30 m from trees and buildings, with the exception of TCNW (~10 m). Two identical bulk collectors set on PVC tubes 1 meter aboveground were established three meters apart at each of the sites (*n* = 12 collectors total). Bulk collectors remain open between sampling periods and primarily collect N ions dissolved in rainwater and some amount of coarse particles heavy enough to settle on collector surfaces between rainfall events [42]. Relative to total dry N deposition, gaseous and particulate N deposition to bulk collectors is often low (e.g., [9]).

Following the design of Simkin et al. [43], bulk collectors consisted of a 20-cm diameter high-density polyethylene funnel attached to Tygon tubing, a HDPE connector, and a chromatograph column filled with 20 mL of ion-exchange resin. Before sampling, all components were rinsed and then soaked in double deionized water for 24 hours. Components were rinsed again and left to dry in a clean enclosed area. In this study, Amberlite IRN 150 mixed-bed ion exchange resin (Rohm & Haas Company, Philadelphia, PA) was used to capture both positively and negatively charged ions as rainfall passed through the collectors [44]. To prepare the resin, double-deionized water was added to the resin to create a slurry. Each column fitted with a 30-μm pore size filter at the bottom was filled with 20 mL of resin. Prepared columns were refrigerated before use.

We evaluated the efficiency of ion capture as well as the extraction efficiency of the resin after Fenn et al. [45]. Columns were loaded with 0, 101, 138, 332, 664, 1665, and 6652 μeq of N as NH_4_Cl and 0, 32, 250, 104, 208, 517, and 2080 μeq of N as KNO_3_. The efficiency of ion capture was >98% for NH_4_^+^ and ~100% for NO_3_^-^. Minimum extraction efficiency, the percentage of ions loaded on the columns and recovered in sequential extractions, was 79% for NH_4_^+^ and 86% for NO_3_^-^. We also used the fixed exchange capacity of the resin (cation = 1.9 eq L^-1^, anion = 1.2 eq L^-1^), rainfall amount, and chemical composition data (2011–2013) from the L.B.J. NADP site to estimate the potential range of cation plus anion loading at our study sites (S1 Text). Based on our calculations and previous studies (e.g., [46]), we determined that resin columns could be collected every three months.

Bulk ion-exchange resin collectors were assembled and installed at the sites on 4 April 2014. A polywool filter was inserted into the neck of each funnel to prevent debris and insects from falling into the resin column. Samples were collected on 11 July 2014, 11 October 2014, 9 January 2015, 10 April 2015, and 13 July 2015. The last columns were collected on 9 October 2015, for a total of six consecutive sampling seasons: Spring 2014 (Apr-Jun; 98 days), Summer 2014 (Jul-Sep; 92 days), Fall 2014 (Oct-Dec; 90 days), Winter 2014 (Jan-Mar; 91 days), Spring 2015 (Apr-Jun; 94 days), and Summer 2015 (Jul-Sep; 88 days). Upon collection, funnels, tubing, connectors, and polywool filters were replaced with new, clean components, and new ion-exchange resin columns were attached. In addition to field samples, two blank resin columns were prepared for each sampling period.

Resin columns were transported to the Ecosystem Geography Laboratory at the University of North Texas where they were refrigerated at 4°C until sample extraction. Bulk deposition and blank samples were extracted three times with 100 mL 2 *M* KCl [27, 44], frozen and then shipped to the Cary Institute of Ecosystem Studies where NH_4_^+^-N and NO_3_^-^-N were determined on a Lachat QuickChem Flow Injection Analyzer +8000 Series (Lachat Instruments, Loveland, Colorado). Ammonium concentrations were determined using the phenate method, while NO_3_^-^-N concentrations were determined using the cadmium diazotization method. Detection limit for both methods was 0.02 mg L^-1^. A series of standards was included with each instrument run.

### Calculation of bulk deposition

Bulk NH_4_^+^-N and NO_3_^-^-N concentrations (mg L^-1^) were multiplied by extractant volume (0.3 L), converted to kg, and then divided by collector surface area to obtain deposition rate (kg ha^-1^ sampling period^-1^). Lab blank concentrations were all below detection limit (<0.02 mg L^-1^). At each site, values for the two bulk collectors were similar (S1 Fig) and averaged for each sampling period. Twelve samples (~15%) were deemed invalid because samplers: were knocked over, had visual evidence of bird droppings and samples with NH_4_^+^-N values more than two times higher than those of the paired collector, were clogged, or were attached to columns with missing labels. In these cases, values from the remaining collector were used. For the NADP L.B.J. site, weekly wet deposition was calculated for each sample period by multiplying NH_4_^+^-N and NO_3_^-^-N concentrations times rainfall amount.

Accumulated deposition per site was calculated by summing the values for the six sample periods. Deposition values were also normalized (i.e., divided) by rainfall amount at each site to assess the relative effect of urban exposure (i.e., urban atmospheric N pollution) on N deposition. To obtain annual N deposition, accumulated deposition over the entire study period was divided by the total number of days sampled (553 days) and the daily deposition value multiplied by 365.

### Phytometer experiment

A phytometer experiment was conducted to investigate the effects of N deposition on plant biomass and tissue quality of two native prairie grass species. Phytometer experiments involve placing plants from one population into multiple locations and then monitoring their growth [47]. The focal plant species were little bluestem (*Schizachyrium scoparium*) and Texas wintergrass *(Nasella leucotricha)*. Little bluestem, a C_4_ warm-season perennial, was selected because of its adaptation to the ecoregion and dominance in the native prairie landscape. Little bluestem begins active growth in late spring and grows through the summer until the first frost in fall [48]. Texas wintergrass is a cool-season C_3_ perennial that thrives in disturbed areas. This species experiences the most rapid growth in early fall and grows through winter and spring [49]. Texas wintergrass was chosen to serve as counterpart to little bluestem for two reasons: (1) C_3_ and C_4_ plants may respond differently to N additions due to varying sensitivity to seasonal water and N availability [50] and (2) it is a little-studied species compared to little bluestem, but one that represents an important component of the Fort Worth prairie. Both little bluestem and Texas wintergrass are bunchgrasses, which reproduce vegetatively via the growth of new tillers from buds on existing tillers. This results in a dense bunch of grass with the oldest tillers towards the center. Sexual reproduction occurs in each grass species with the formation of specialized flowering tillers that are wind pollinated.

All plants for the phytometer experiment were obtained from one population grown at a local nursery in Mansfield, Texas. Clumps of little bluestem were divided into smaller clumps, each possessing 1–4 tillers and weighing between 0.5 g and 2 g. Texas wintergrass plants were divided into clumps of 2–10 tillers each and similarly weighed between 0.3 g and 1.5 g. Divided clumps were potted individually in black plastic pots (3785 cm^3^) with weed barrier on the bottom and sandy loam topsoil sourced from Mansfield.

A total of 20 little bluestem and 20 Texas wintergrass plants were randomly assigned to each of the N deposition measurement sites. Adjacent to the bulk deposition collectors, black weed barrier was attached to the ground in a 6 x 7.5 m rectangle to prevent competition from established plants and minimize weed invasion. A water barrel was installed at each site to facilitate watering. Little bluestem plants were placed at the sites between 27–29 September 2013, while Texas wintergrass plants were placed at the sites on 5–6 April 2014. During the experiment, all plants grew in full sun and were watered biweekly with 500 ml of water per pot. Local potable water was used and therefore did not represent a source of nitrogen for the plants. Little bluestem plants were harvested on 22 July 2014. Due to human disturbance and significant loss of plants at three sites, data for little bluestem are presented for the ALVA, TCNW, and TCSO sites only. Texas wintergrass plants were harvested between 20 May and 1 June 2015.

Following harvest, plants were returned to the lab where they were separated into tillers (vegetative and reproductive) and roots, dried at 52°C for 72 hours, and then weighed to obtain tiller (hereafter shoot) and root biomass. Biomass values were divided by the total number of days plants were in the ground to enable species comparisons. Subsamples (~5–10 g) of shoot and root biomass were ground using a Wiley Mill, weighed, placed in a tin capsule, and analyzed for total C and N on a Perkin-Elmer 2400 CHN analyzer. Total plant (shoot + root) N for little bluestem and Texas wintergrass was estimated by multiplying shoot and root biomass by percent N of each and then summing the values.

### Statistical analyses

All variables were tested for normality using the Shapiro-Wilk test. Variables with non-normal distributions were log-transformed to meet assumptions of normality and homogeneity of variance. Nonparametric tests were used when sample data did not meet these assumptions.

We examined relationships of urban development with N deposition using simple linear regressions, and we tested for seasonal differences in N deposition using the Kruskal-Wallis Test with the Dunn Method for joint ranking. For each of the plant traits measured, t-tests were used to examine contrasts between species within each of the three sites (ALVA, TCNW, TCSO) along the gradient where both species were present. Site-to-site differences in little bluestem and Texas wintergrass traits were assessed using the Kruskal-Wallis test followed by the Steel-Dwass test for multiple comparisons. Plant trait relationships with N deposition from planting date to harvest date were also evaluated with simple linear regression. All statistical analyses were performed in JMP v.14 [51]. Significance was set at *p* < 0.1.

## Results

### Nitrogen deposition along an urban development gradient

Bulk inorganic N deposition measured in DFW ranged from 6.1 to 9.9 kg ha^-1^ yr^-1^ across sites (Table 1). Sites with ≥15% urban land received 30–100% more N than the L.B.J. reference site (5 kg ha^-1^ yr^-1^). Ammonium-N was higher than NO_3_^-^-N deposition at all sites and during all seasons sampled. Ammonium-N constituted 63–70% of inorganic N.

**Fig 2:**
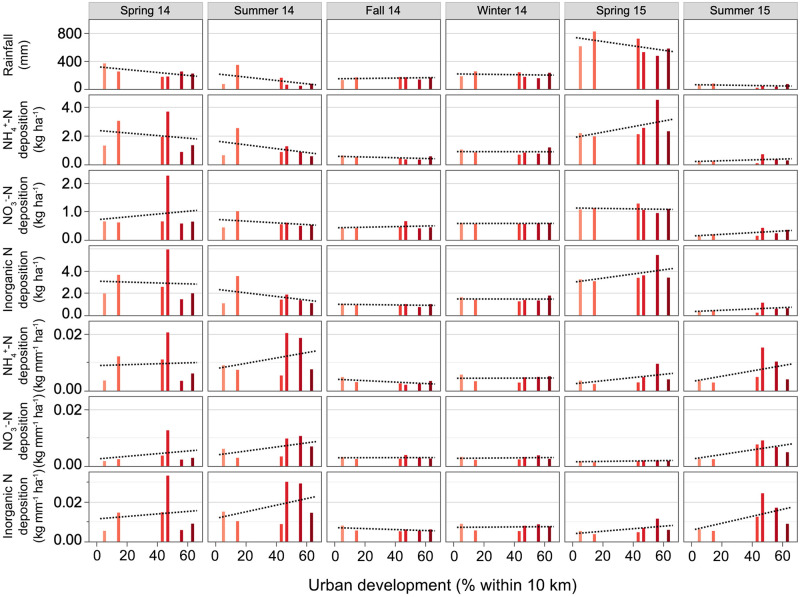
Relationships between urban development, rainfall, and absolute and precipitation-normalized bulk nitrogen deposition for six consecutive sampling seasons. Nitrogen deposition was sampled in Spring 2014 (Apr-Jun), Summer 2014 (Jul-Sep), Fall 2014 (Oct-Dec), Winter 2014 (Jan-Mar), Spring 2015 (Apr-Jun), and Summer 2015 (Jul-Sep). Percent urban development within 10 km of each site in the Dallas-Fort Worth metropolitan area ranges from low (light pink) to high (crimson red).

Urban development was not correlated with absolute or normalized bulk NH_4_^+^-N or inorganic N deposition (Fig 2; S1 Table). Nitrate-N deposition also did not increase with proportion of urban land (Fig 2). When normalized by rainfall, NO_3_^-^-N deposition increased along the urban development gradient in Spring 2015 (p = 0.12) and Summer 2015 (p = 0.13), but the relationships were not significant.

Significant seasonal effects on N deposition were evident, with peaks in spring. In the spring of 2014 and 2015, bulk NH_4_^+^-N deposition was 4-6-fold higher compared to Fall 2014 and 6-9-fold higher compared to Summer 2015. Similarly, in both years, Spring NO_3_^-^-N deposition was 4–5 times the level measured in Summer 2015, and Spring 2015 NO_3_^-^-N deposition was double that in Fall 2014. Nitrate-N deposition in Winter 2014 was also 2.5-fold higher than in Summer 2015. Seasonal differences in inorganic N deposition were identical to those of NH_4_^+^-N deposition.

### Little bluestem and Texas wintergrass plant traits

**Fig 3:**
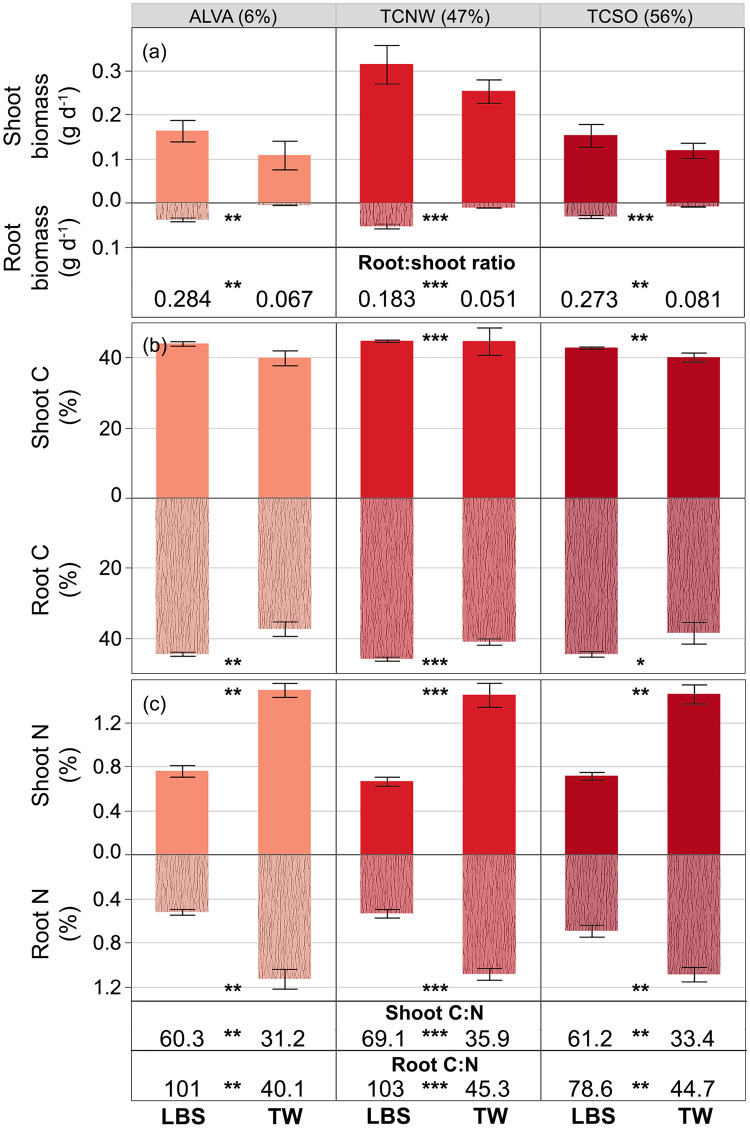
Little bluestem (LBS) and Texas wintergrass (TW) plant traits. Differences in (a) mean (±1SE) biomass accumulation, allocation, (b) tissue C, and (c) tissue N between native prairie grass species at three sites characterized by low (pink) to high (crimson red) urban development in the Dallas-Fort Worth metropolitan area. *p < 0.1, ** *p* < 0.05, *** *p* < 0.001.

Little bluestem and Texas wintergrass plants growing at ALVA, TCNW, and TCSO differed in their root biomass accumulation, biomass allocation, tissue C and N, and total plant N. Shoot biomass was similar for both species, but little bluestem accumulated four to eight times more root biomass than Texas wintergrass, resulting in higher mean root:shoot ratios for little bluestem (Fig 3a). Shoot and root C differed significantly but less markedly between species (Fig 3b) than did shoot and root N. Shoot and root N were approximately two-fold higher in Texas wintergrass compared to little bluestem (Fig 3c). Given the higher tissue N, Texas wintergrass had lower tissue C:N ratios than little bluestem across all sites and higher overall total plant N at the two more urban sites (Table 2).

**Table 2:** Plant traits (mean±1SE) for the six phytometer sites along an urban development gradient (indicated in parentheses).

	ALVA (6%)	DENT (15%)	LLELA (44%)	TCNW (47%)	TCSO (56%)	TCNE (64%)
	Little bluestem (*Schizachyrium scoparium*)					
	*n* = 10			*n* = 10	*n* = 10	
Shoot biomass (g d^-1^)	0.16±0.02^b^	-	-	0.32±0.04^a^	0.15±0.03^b^	-
Root biomass (g d^-1^)	0.04±0.004^ab^	-	-	0.05±0.005^a^	0.03±0.004^b^	-
Total biomass (g d^-1^)	0.20±0.03^b^	-	-	0.37±0.05^a^	0.18±0.03^b^	-
Root:shoot	0.28±0.05^a^	-	-	0.18±0.01^a^	0.27±0.05^a^	-
Shoot C (%)	44.0±0.64^ab^	-	-	44.7±0.37^a^	42.8±0.32^b^	-
Shoot N (%)	0.76±0.05^a^	-	-	0.67±0.04^a^	0.72±0.04^a^	-
Shoot C:N	60.3±4.27^a^	-	-	69.1±3.76^a^	61.2±3.41^a^	-
Root C (%)	44.5±0.58^a^	-	-	45.9±0.59^a^	44.5±0.78^a^	-
Root N (%)	0.52±0.03^b^	-	-	0.54±0.04^b^	0.70±0.05^a^	-
Root C:N	101±5.99^a^	-	-	103±5.89^a^	78.6±5.95^b^	-
Total plant N (g)	0.42±0.05^b^	-	-	0.72±0.10^a^	0.37±0.05^b^	-
	Texas wintergrass (*Nasella leucotricha*)					
	*n* = 10	*n* = 15	*n* = 15	*n* = 19	*n* = 14	*n* = 14
Shoot biomass (g d^-1^)	0.11±0.03^b^	0.16±0.02^ab^	0.08±0.01^b^	0.25±0.03^a^	0.12±0.02^b^	0.08±0.01^b^
Root biomass (g d^-1^)	0.005±0.001^c^	0.01±0.001^bc^	0.005±0.001^c^	0.01±0.001^a^	0.01±0.001^ab^	0.005±0.001^c^
Total biomass (g d^-1^)	0.11±0.03^b^	0.17±0.02^ab^	0.08±0.01^b^	0.26±0.03^a^	0.13±0.02^b^	0.09±0.01^b^
Root:shoot	0.07±0.011^ab^	0.05±0.005^b^	0.08±0.012^ab^	0.05±0.005^b^	0.08±0.007^a^	0.05±0.004^b^
Shoot C (%)	39.9±2.1^ab^	42.4±1.1^a^	38.4±1.1^b^	44.6±3.9^a^	40.1±1.3^ab^	42.3±0.6^a^
Shoot N (%)	1.50±0.06^a^	1.21±0.06^bc^	1.20±0.06^bc^	1.46±0.11^ab^	1.46±0.09^ab^	1.25±0.05^bc^
Shoot C:N	31.2±1.7^b^	41.6±1.7^a^	38.5±2.0^ab^	35.9±1.1^ab^	33.4±2.3^b^	40.2±1.9^a^
Root C (%)	37.4±2.1^b^	42.1±0.9^ab^	42.0±0.4^a^	41.0±0.9^ab^	38.5±3.1^ab^	43.0±0.6^a^
Root N (%)	1.13±0.09^ab^	0.98±0.05^bc^	0.85±0.04^c^	1.09±0.05^ab^	1.09±0.07^ab^	1.29±0.05^a^
Root C:N	40.1±3.0^bc^	51.4±1.8^ab^	59.4±2.8^a^	45.3±1.9^bc^	44.7±1.4^bc^	39.4±1.3^c^
Total plant N (g)	0.69±0.20^b^	0.86±0.13^ab^	0.41±0.06^b^	1.54±0.17^a^	0.73±0.09^b^	0.42±0.04^b^

Different letters denote statistically significant differences among sites (*p* < 0.1).

Hyphen (-) indicates missing data due to human disturbance and significant loss of plants.

### Prairie grass responses to nitrogen deposition

Both little bluestem and Texas wintergrass plants exhibited site-to-site differences in plant traits (Table 2). All measures of little bluestem biomass were highest at TCNW and lowest at TCSO. At harvest, plants at TCNW had twice the shoot and total biomass as plants at the other sites. Root C:N was also lowest at TCSO due to elevated root N compared to the other sites. Although little bluestem plants at TCNW did not have higher shoot or root N compared to the other sites, total plant N was nearly double that measured at other sites there given the higher total plant biomass.

Similar to little bluestem, Texas wintergrass plants accumulated the most shoot, root, and total biomass at TCNW, as much as three times more than plants at the other sites. Plants grown at TCSO had the highest root:shoot ratio of all sites. Although patterns for tissue C and N were less clear, the highest shoot C, shoot C:N, root C, and root N were recorded at TCNE, the most urban site.

**Fig 4:**
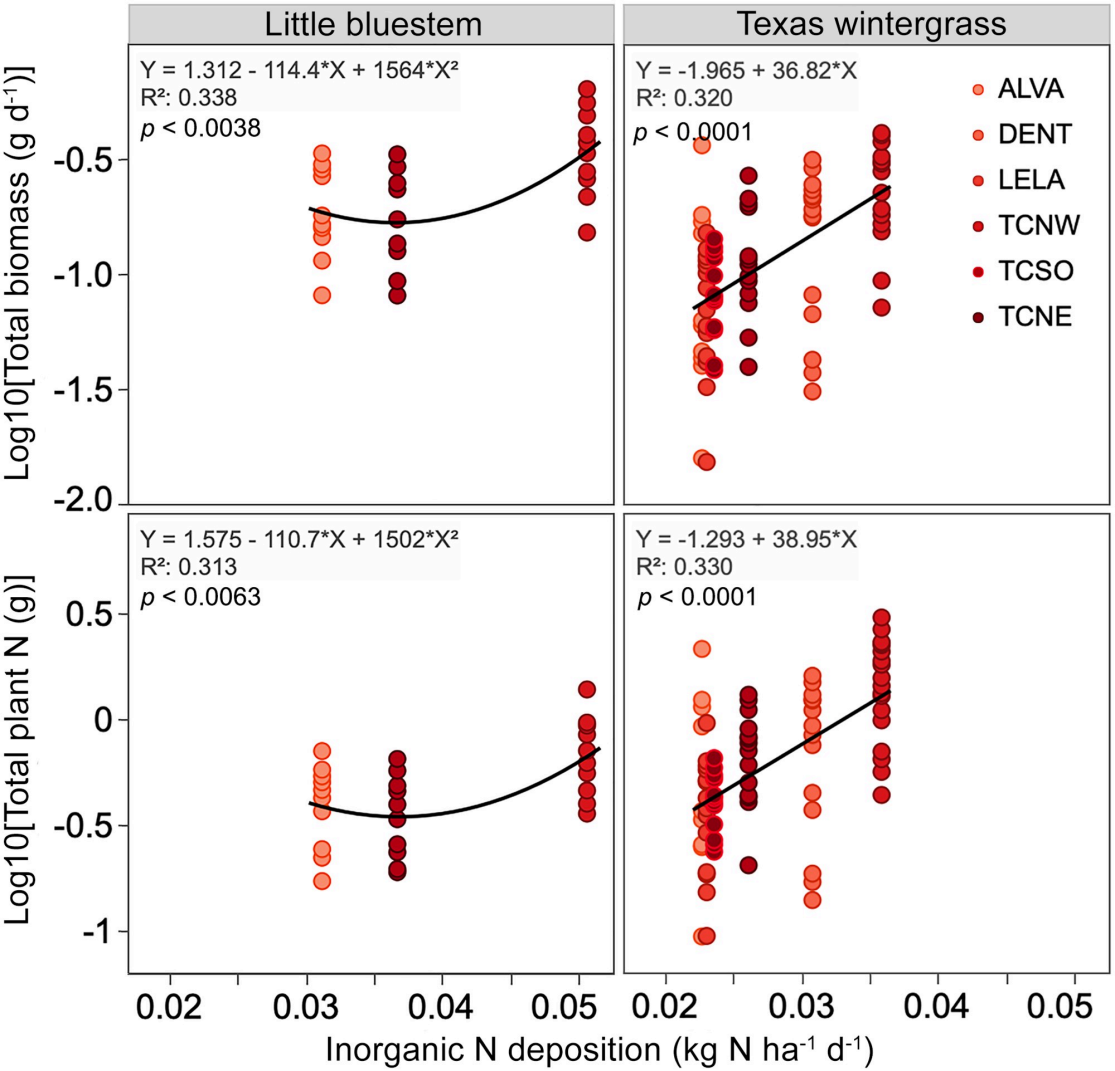
Prairie grass responses to nitrogen deposition. Increase in total plant biomass (g d^-1^) and total plant N (g) with inorganic nitrogen deposition (kg ha^-1^ d^-1^) from planting date to harvest date for little bluestem and Texas wintergrass plants at six phytometer sites in the Dallas-Fort Worth metropolitan area.

Both species showed a significant increase in total biomass (Fig 4) and total plant N with inorganic N deposition (Fig 4; S2 Table). For Texas wintergrass, changes in total biomass and plant N were most pronounced at sites receiving >0.025 kg ha^-1^ d^-1^ of inorganic N (~9 kg ha^-1^ yr^-1^).

## Discussion

### Nitrogen deposition in Dallas-Fort Worth: Magnitude, seasonality, and form

Here we provide a first estimate of inorganic N deposition in Dallas-Fort Worth, the fourth largest U.S. metropolitan area and a major node in the Texas Triangle megapolitan area. We find that bulk inorganic N deposition in this semi-arid city is relatively high, 6.1–9.9 kg ha^-1^ yr^-1^, and more comparable to bulk N deposition measured in wetter cities such as Boston (5.2 kg ha^-1^ yr^-1^; [52]) than in arid cities such as Phoenix (1.9 kg ha^-1^ yr^-1^; [9]).

Moreover, N deposition in DFW varies seasonally with lower rates in fall and pulsed deposition in spring. Several factors contribute to temporal variability in urban atmospheric wet deposition, including changes in the source and strength of emissions, meteorological conditions, and the rate of chemical reactions in the atmosphere (e.g., [9, 53–56]). At our study sites, 43% of NH_4_^+^-N, 34% of NO_3_^-^-N, and 40% of the bulk inorganic N measured between April 2014 and March 2015 occurred in spring compared to 30% of rainfall, suggesting that both increased rainfall and N concentrations contributed to elevated deposition during this season. In 2015, we captured a month of record-breaking rainfall, in which DFW received nearly half its mean annual rainfall in May alone. As a result, spring N deposition was 1.5 to 4.5-fold higher compared to Spring 2014 depending on the site. Long-term data from the L.B.J. National Grasslands NADP site confirm that spring deposition peaks are typical for this region. From 1983 to 2019, spring accounted for 30% of annual rainfall and 40% of inorganic N at L.B.J. [40].

During all seasons and across all sites, the majority of N deposited was in the form of NH_4_^+^-N (63–70%). This is in line with previous studies that show NH_4_^+^-N comprising 70–77% of bulk inorganic N in Boston [6, 8], 69% of wet deposition in Providence [57], ~53% in Phoenix [9], ~59% of wet inorganic N deposition across the continental U.S. [58], and 60% of N in urban areas globally [3]. The dominance of NH_4_^+^-N in the DFW area is consistent with an observed shift over time from NO_3_^-^- to NH_4_^+^-dominated N deposition in U.S. urban areas likely resulting from increased NO_x_ regulations and lack of NH_3_ regulations [8, 58, 59], as well as NO_x_ reduction technologies that produce NH_3_ [60].

Myriad local emission sources contribute to elevated NH_4_^+^-N deposition within urban areas. Lawn and greenspace fertilizer application [8, 53], industrial and fossil fuel combustion [58] and wastewater treatment [61] represent important sources of gaseous ammonia to the atmosphere. Off-site emissions from crop and livestock production also influence urban N deposition [9]. In a recent study, measured and estimated on-road and agricultural NH_3_ emissions in Dallas and Tarrant counties––where the cities of Dallas and Fort Worth are located––were notably high [58]. Although we cannot apportion sources of N at our study sites, high levels of traffic congestion [62], intensive and extensive landscaping [63], and growing proportions of land under agriculture [64] likely contribute to the prevalence of NH_4_^+^-N deposition in DFW.

### Urban development effects on N deposition

Bulk inorganic N deposition in DFW was high compared to the L.B.J. reference site but did not, contrary to our hypothesis, increase with urban development within 10 km of the sites. We expected such a relationship given previous studies in arid cities, which show positive relationships between N deposition and urbanization proxies. For example, at sites in the Denver-Boulder metropolitan area and at oak savanna sites in central California, either bulk N concentrations or deposition exhibited positive relationships with population density [56] and proximity to urban core [55]. In contrast, Lohse et al. [54] found that neither distance to urban core nor land use were related to rainwater N concentrations, while Cook et al. [9] found little variation in bulk N deposition along an urban-rural gradient in the Phoenix metropolitan area.

There are several possible reasons why we did not detect a relationship between annual N deposition and urban development. First, estimates indicate that 74–84% and 51–61% of total (wet + dry) inorganic N deposition to our study sites in 2014 and 2015 [65, 66], respectively, was in the form of dry deposition. Although bulk collectors capture varying amounts of dry N deposition [67, 68], this amount is likely low relative to total dry inputs (e.g., [54]). Second, urban land within 10 km may not serve a good proxy for NH_4_^+^-N or NO_3_^-^-N deposition. Strong concentration gradients in atmospheric N due to localized emissions near roads and lower emissions in park areas [69] may have obscured spatial variation in N deposition. For example, one of the more urban (44%) sites sampled in this study was located within a designated natural area (LLELA) and had comparatively low deposition compared to most of the other sites. Gaseous ammonia has a short atmospheric lifetime (on the order of hours) and tends to deposit close to the source [70]. Thus, the location of this site relative to emissions source (e.g., roads) may have affected deposition rates there. In the case of NO_3_^-^-N, the longer atmospheric lifetime (~1 day; [71]) and potential for long-range transport of NO_x_ in the troposphere [72] might prevent us from observing relationships between deposition and urban development within a 10-km buffer. Third, variation in atmospheric mixing processes due to variability in wind speed, surface roughness, and heat island effects could have contributed to heterogeneous patterns of N deposition [69].

### Prairie grass responses to N deposition

Although the relationship between N deposition and level of urban development was not what we anticipated, our findings show that two common native prairie grass species in the Southern Great Plains exhibit a positive biomass response, and in turn higher plant N, with greater N deposition. This response is consistent with previous research indicating that N additions increase grassland primary productivity [73–75] and the expectation that as N becomes less limiting, plants allocate a greater proportion of biomass aboveground. In a global meta-analysis of 42 plots in the Nutrient Network Global Research Cooperative (http://www.nutnet.unm.edu), where N deposition ranged from <1 to 36 kg N ha^-1^ yr^-1^, Stevens et al. [75] found a 3% increase in aboveground net primary production with each additional unit of N deposition.

In this study, sites receiving 7.2–9.9 kg inorganic N ha^-1^ yr^-1^, the high end of the deposition range we found, exhibited the most pronounced changes in total biomass and plant N. As these values do not include dry deposition, we extracted wet and dry N deposition (2014–2015) from NADP total deposition maps. NADP total deposition estimates were developed using a hybrid approach that combines measured and modeled data [65]. The estimates indicate that our DFW sites receive a total (wet + dry) N deposition load of 11–13 kg N ha^-1^ yr^-1^. If we sum bulk deposition measured in this study with estimates of dry deposition from NADP total deposition maps, N deposition rates are even higher, 13–18 kg N ha^-1^ yr^-1^. Thus, native prairie grasses may exhibit a positive biomass response to increased urban N deposition up to ~18 kg ha^-1^ yr^-1^. Moreover, both ranges of total inorganic N deposition surpass the critical load limit for herbaceous plant species richness in the DFW area, which we estimated to be 8.9 kg N ha^-1^ yr^-1^ after Simkin et al. [20]. Nevertheless, larger scale and longer-term monitoring in the DFW are needed to assess the effects of N deposition on shifts in plant community composition.

### Implications for native prairie grass species

At levels above N critical loads, grassland ecosystems experience changes in species composition which may include loss of N-sensitive and native species, shifts in dominance, and decreased species richness [18, 20, 76, 77]. Thus, increased rates of ambient urban N deposition could affect native prairie grass remnants in the Southern Great Plains. Consistent with previous studies [78, 79], little bluestem plants in our phytometer experiment had low tissue N compared to Texas wintergrass but allocated a higher proportion of biomass belowground. The high N-use efficiency and more extensive root network of little bluestem are traits that make this species a good competitor on N-poor soils [79]. In contrast, Texas wintergrass had higher tissue N but invested less in root biomass, potentially indicating a higher N uptake rate [80].

Texas wintergrass is unique among cool-season grasses in that its range is restricted south of 35° N latitude [81], yet it is an important component of the prairie in the Southern Great Plains. We know of only one study in which little bluestem and Texas wintergrass responses to light and nitrogen were assessed, both in monoculture and in mixture [81]. In that study, both species grew best under high light and high N––similar to our phytometer experiment in which both species accumulated the most biomass at the highest N deposition site. However, Texas wintergrass accumulated more biomass when grown in mixture, suggesting that this species may be able to better use additional N in a community setting. Little bluestem, on the other hand, is vulnerable to loss at high N levels [76]; a recent study reported a N critical load range of 9–14 kg ha^-1^ yr^-1^ for this species [18]. Using data specific to our study area and the model presented by Clark et al. [18], the critical load for little bluestem in DFW is 13.9 kg ha^-1^ yr^-1^. Little bluestem thus appears to be near or at the critical load limit for N, meaning this species is potentially vulnerable to loss.

Future work is needed to determine the effect of N on these species especially in the context of Great Plains drought [82], precipitation variability [83], and urban pollution and heat effects. In the Southern Great Plains ecoregion, little bluestem is the dominant while Texas wintergrass is a common component of late-successional tallgrass prairie communities. Loss of one or both of these species could therefore have significant negative implications for plant community composition and higher trophic levels. As urbanization continues to have multiple effects on local vegetation, the role of N deposition in affecting individual species and communities warrants additional study.

## Supporting information

S1 Table Results of regression analyses, including regression coefficient, standard error, p-values, and goodness of fit. (DOCX)

S2 Table Results of regression analyses, including regression coefficient, standard error, p-values, and goodness of fit. (DOCX)

S1 Text Estimates of the potential range of cation plus anion loading. (DOCX)

S1 Fig Nitrogen deposition rates per collection period for both samplers at each of the six urban sites sampled in the Dallas-Fort Worth metropolitan area from April 2014 to October 2015. (DOCX)
