# Supplementary material for: Ambient urban N deposition drives increased biomass and total plant N in two native prairie grass species in the U.S. Southern Great Plains
Source: PLoS One. 2021 May 6;16(5):e0251089. doi: 10.1371/journal.pone.0251089 (PMC8101712; doi:10.1371/journal.pone.0251089)
Supplement: S1 Table — (DOCX) [file pone.0251089.s001.docx]

**S1 Table**

Simple linear regression results for Figure 2.

Dependent variable: Rainfall

| Variable | Coefficient | Std Error | P value | R^2^ |
| --- | --- | --- | --- | --- |
| Urban development |  |  |  |  |
| Spring 2014 | -2.0610 | 1.0967 | 0.1334 | 0.469 |
| Summer 2014 | -2.3857 | 2.1432 | 0.3280 | 0.237 |
| Fall 2014 | 0.2597 | 0.3761 | 0.5279 | 0.107 |
| Winter 2014 | -0.2669 | 0.8579 | 0.7712 | 0.024 |
| Spring 2015 | -3.1200 | 2.2781 | 0.2427 | 0.319 |
| Summer 2015 | -0.3080 | 0.4627 | 0.5421 | 0.100 |

Dependent variable: Ammonium-N Deposition (kg ha^-1^)

| Variable | Coefficient | Std Error | P value | R^2^ |
| --- | --- | --- | --- | --- |
| Urban development |  |  |  |  |
| Spring 2014 | -0.0090 | 0.0232 | 0.7182 | 0.036 |
| Summer 2014 | -0.0133 | 0.0142 | 0.4017 | 0.180 |
| Fall 2014 | -0.0026 | 0.0021 | 0.2857 | 0.275 |
| Winter 2014 | -0.0002 | 0.0040 | 0.9585 | 0.001 |
| Spring 2015 | 0.0195 | 0.0179 | 0.3381 | 0.228 |
| Summer 2015 | -0.0030 | 0.0043 | 0.5334 | 0.104 |

Dependent variable: Nitrate-N Deposition (kg ha^-1^)

| Variable | Coefficient | Std Error | P value | R^2^ |
| --- | --- | --- | --- | --- |
| Urban development |  |  |  |  |
| Spring 2014 | 0.0050 | 0.0141 | 0.7429 | 0.030 |
| Summer 2014 | -0.0031 | 0.0042 | 0.5027 | 0.119 |
| Fall 2014 | 0.0010 | 0.0020 | 0.6284 | 0.064 |
| Winter 2014 | 0.0000 | 0.0003 | 0.9214 | 0.003 |
| Spring 2015 | -0.0007 | 0.0023 | 0.7616 | 0.026 |
| Summer 2015 | 0.0030 | 0.0020 | 0.2080 | 0.360 |

Dependent variable: Inorganic N Deposition (kg ha^-1^)

| Variable | Coefficient | Std Error | P value | R^2^ |
| --- | --- | --- | --- | --- |
| Urban development |  |  |  |  |
| Spring 2014 | -0.0040 | 0.0355 | 0.9151 | 0.003 |
| Summer 2014 | -0.0163 | 0.0182 | 0.4212 | 0.167 |
| Fall 2014 | -0.0015 | 0.0022 | 0.5257 | 0.108 |
| Winter 2014 | -0.0002 | 0.0042 | 0.9666 | 0.001 |
| Spring 2015 | 0.0187 | 0.0162 | 0.3132 | 0.249 |
| Summer 2015 | 0.0059 | 0.0061 | 0.3903 | 0.188 |

Dependent variable: Normalized Ammonium-N Deposition (kg mm^-1^ ha^-1^)

| Variable | Coefficient | Std Error | P value | R^2^ |
| --- | --- | --- | --- | --- |
| Urban development |  |  |  |  |
| Spring 2014 | 0.0000 | 0.0001 | 0.9136 | 0.003 |
| Summer 2014 | 0.0001 | 0.0001 | 0.5063 | 0.117 |
| Fall 2014 | -0.0000 | 0.0000 | 0.1944 | 0.377 |
| Winter 2014 | 0.0000 | 0.0000 | 0.9544 | 0.001 |
| Spring 2015 | 0.0001 | 0.0000 | 0.3022 | 0.259 |
| Summer 2015 | 0.0001 | 0.0001 | 0.3775 | 0.197 |

Dependent variable: Normalized Nitrate-N Deposition (kg mm^-1^ ha^-1^)

| Variable | Coefficient | Std Error | P value | R^2^ |
| --- | --- | --- | --- | --- |
| Urban development |  |  |  |  |
| Spring 2014 | 0.0000 | 0.0001 | 0.6138 | 0.069 |
| Summer 2014 | 0.0001 | 0.0001 | 0.2808 | 0.280 |
| Fall 2014 | 0.0000 | 0.0000 | 0.9294 | 0.002 |
| Winter 2014 | 0.0000 | 0.0000 | 0.7537 | 0.028 |
| Spring 2015 | 0.0000 | 0.0000 | 0.1244 | 0.485 |
| Summer 2015 | 0.0001 | 0.0000 | 0.1288 | 0.477 |

Dependent variable: Normalized Inorganic N Deposition (kg mm^-1^ ha^-1^)

| Variable | Coefficient | Std Error | P value | R^2^ |
| --- | --- | --- | --- | --- |
| Urban development |  |  |  |  |
| Spring 2014 | 0.0001 | 0.0002 | 0.7889 | 0.020 |
| Summer 2014 | 0.0002 | 0.0002 | 0.4151 | 0.171 |
| Fall 2014 | -0.0000 | 0.0000 | 0.2441 | 0.318 |
| Winter 2014 | 0.0000 | 0.0000 | 0.8719 | 0.007 |
| Spring 2015 | 0.0001 | 0.0000 | 0.2694 | 0.291 |
| Summer 2015 | 0.0002 | 0.0001 | 0.2486 | 0.313 |
