# Supplementary material for: Ambient urban N deposition drives increased biomass and total plant N in two native prairie grass species in the U.S. Southern Great Plains
Source: PLoS One. 2021 May 6;16(5):e0251089. doi: 10.1371/journal.pone.0251089 (PMC8101712; doi:10.1371/journal.pone.0251089)
Supplement: S2 Table — (DOCX) [file pone.0251089.s002.docx]

**S2 Table**

Regression results for Figure 4.

**Little bluestem**

Dependent variable: Total biomass (g d^-1^)

| Variable | Coefficient1 | Coefficient2 | Std Error | P value | R^2^ |
| --- | --- | --- | --- | --- | --- |
| Inorganic N deposition (kg ha^-1^ d^-1^) | 114.4 | 1564 | 1049 | <0.0038 | 0.338 |

Dependent variable: Total plant N (g)

| Variable | Coefficient1 | Coefficient2 | Std Error | P value | R^2^ |
| --- | --- | --- | --- | --- | --- |
| Inorganic N deposition (kg ha^-1^ d^-1^) | 110.7 | 1502 | 1012 | <0.0063 | 0.313 |

**Texas wintergrass**

Dependent variable: Total biomass (g d^-1^)

| Variable | Coefficient | Std Error | P value | R^2^ |
| --- | --- | --- | --- | --- |
| Inorganic N deposition (kg ha^-1^ d^-1^) | 36.8198 | 5.8282 | <0.0001 | 0.320 |

Dependent variable: Total plant N (g)

| Variable | Coefficient | Std Error | P value | R^2^ |
| --- | --- | --- | --- | --- |
| Inorganic N deposition (kg ha^-1^ d^-1^) | 38.9550 | 6.1232 | <0.0001 | 0.330 |
