# Supplementary material for: Ambient urban N deposition drives increased biomass and total plant N in two native prairie grass species in the U.S. Southern Great Plains
Source: PLoS One. 2021 May 6;16(5):e0251089. doi: 10.1371/journal.pone.0251089 (PMC8101712; doi:10.1371/journal.pone.0251089)
Supplement: S1 Text — (DOCX) [file pone.0251089.s003.docx]

**S1 Text**

Estimates of the potential range of cation plus anion loading at our study sites.

1. Calculate capacity for a liter of resin in which we assume equal amounts of anion and cation resin beads in mixture
2. Estimate capacity for 20 ml of resin within a chromatograph column
3. Download seasonal Precipitation-Weighted Mean Concentrations for nearest NADP site (TX56)

- <https://nadp.slh.wisc.edu/data/sites/siteDetails.aspx?net=NTN&id=TX56>

1. Calculate annual loading rates and approximate saturations for 2011-2013
2. Examine rainfall amounts for sample years and multiply loading rates by a factor to account for potential differences in rainfall between years with historical data and sample years.
