# Supplementary material for: Ambient urban N deposition drives increased biomass and total plant N in two native prairie grass species in the U.S. Southern Great Plains
Source: PLoS One. 2021 May 6;16(5):e0251089. doi: 10.1371/journal.pone.0251089 (PMC8101712; doi:10.1371/journal.pone.0251089)

**S1 Fig**

Nitrogen deposition rates per collection period for both samplers at each of six urban sites sampled in the Dallas-Fort Worth metropolitan area from April 2014 to October 2015.


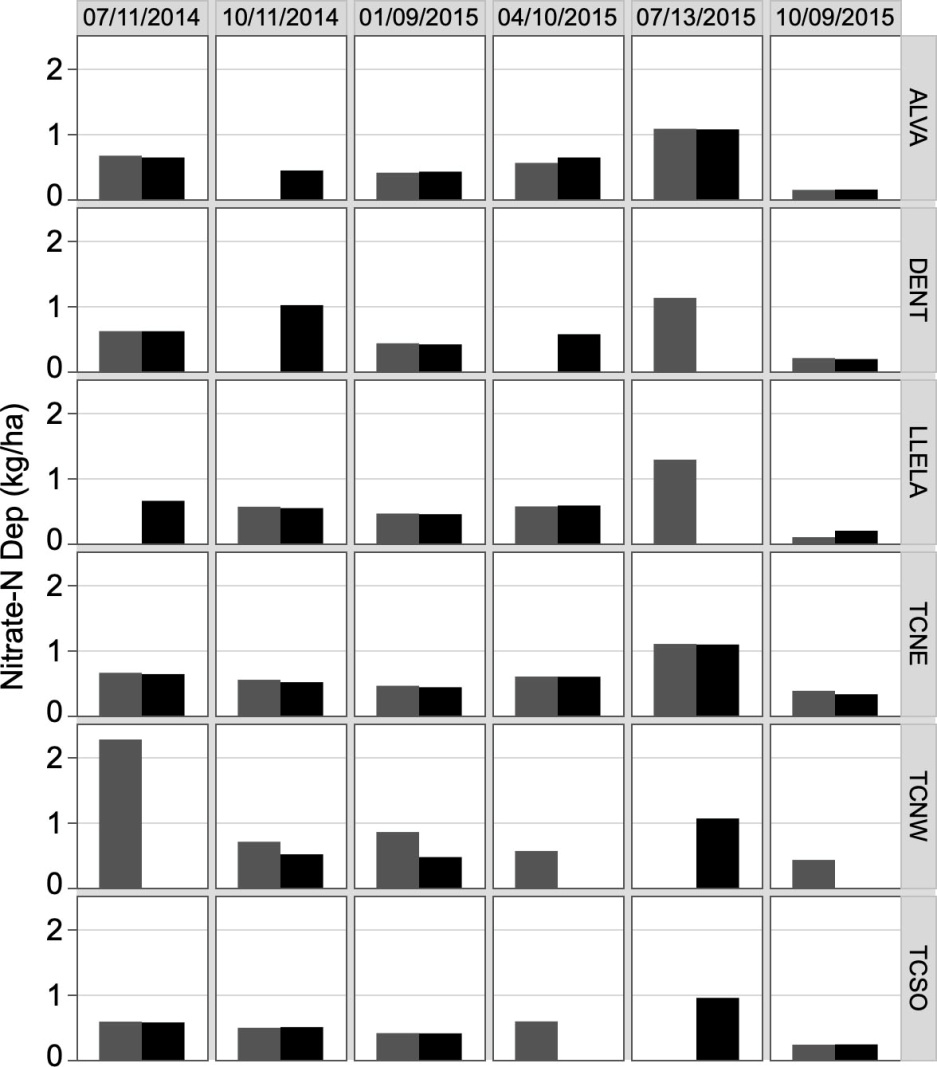

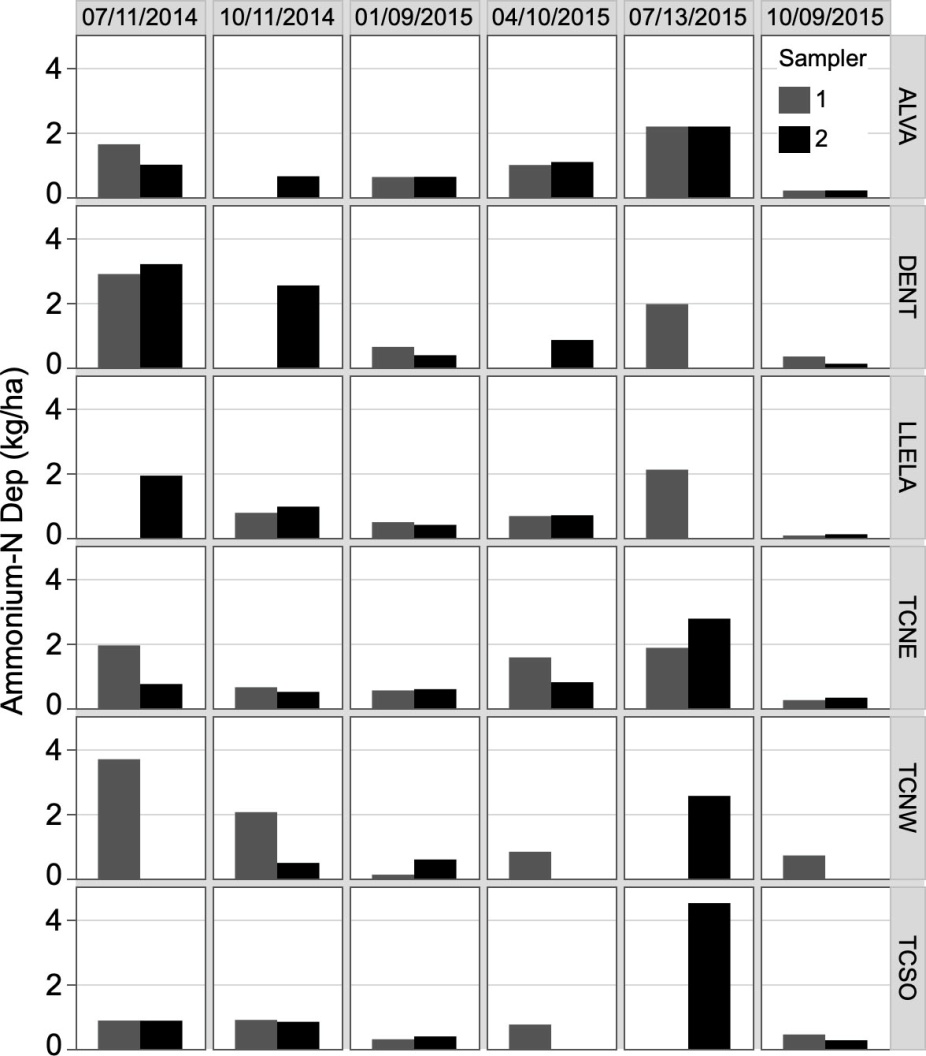

Supplement: S1 Fig — (DOCX) [file pone.0251089.s004.docx]
